# The *C. elegans* pseudogene *sspt-16 (F55A3.7)* is required to safeguard germ cells against reprogramming

**DOI:** 10.17912/micropub.biology.000392

**Published:** 2021-05-18

**Authors:** Andreas Ofenbauer, Clara Maria Kraus, Baris Tursun

**Affiliations:** 1 Berlin Institute of Medical Systems Biology, 10115 Berlin, Germany; 2 Max Delbrück Center for Molecular Medicine in the Helmholtz Association, 13125 Berlin, Germany

## Abstract

We recently identified FAcilitates Chromatin Transcription (FACT) as a reprogramming barrier of transcription factor (TF) mediated conversion of germ cells into neurons in *C. elegans*. FACT is a conserved heterodimer consisting of SPT16 and SSRP1 in mammals. Duplication events during evolution in *C. elegans* generated two SSRP1 homologs named HMG-3 and HMG-4, while SPT-16 is the only homolog of SPT16. Yet, the pseudogene *F55A3.7* has nearly complete nucleotide sequence homology to the *spt-16* gene. However, *F55A3.7* lacks some *spt-16* exons and DNA pieces so we named it *sspt-16* (short *spt-16*)*. *Surprisingly, the deletion mutant *ok1829*, which affects only the *sspt-16* pseudogene, shows similar germ cell reprogramming effects as described previously for FACT-depleted animals. We examined whether lack of *sspt-16* affects other genes or chromatin accessibility, which may explain the permissiveness for germ cell reprogramming.

**Figure 1. sspt-16 is a novel ncRNA:**
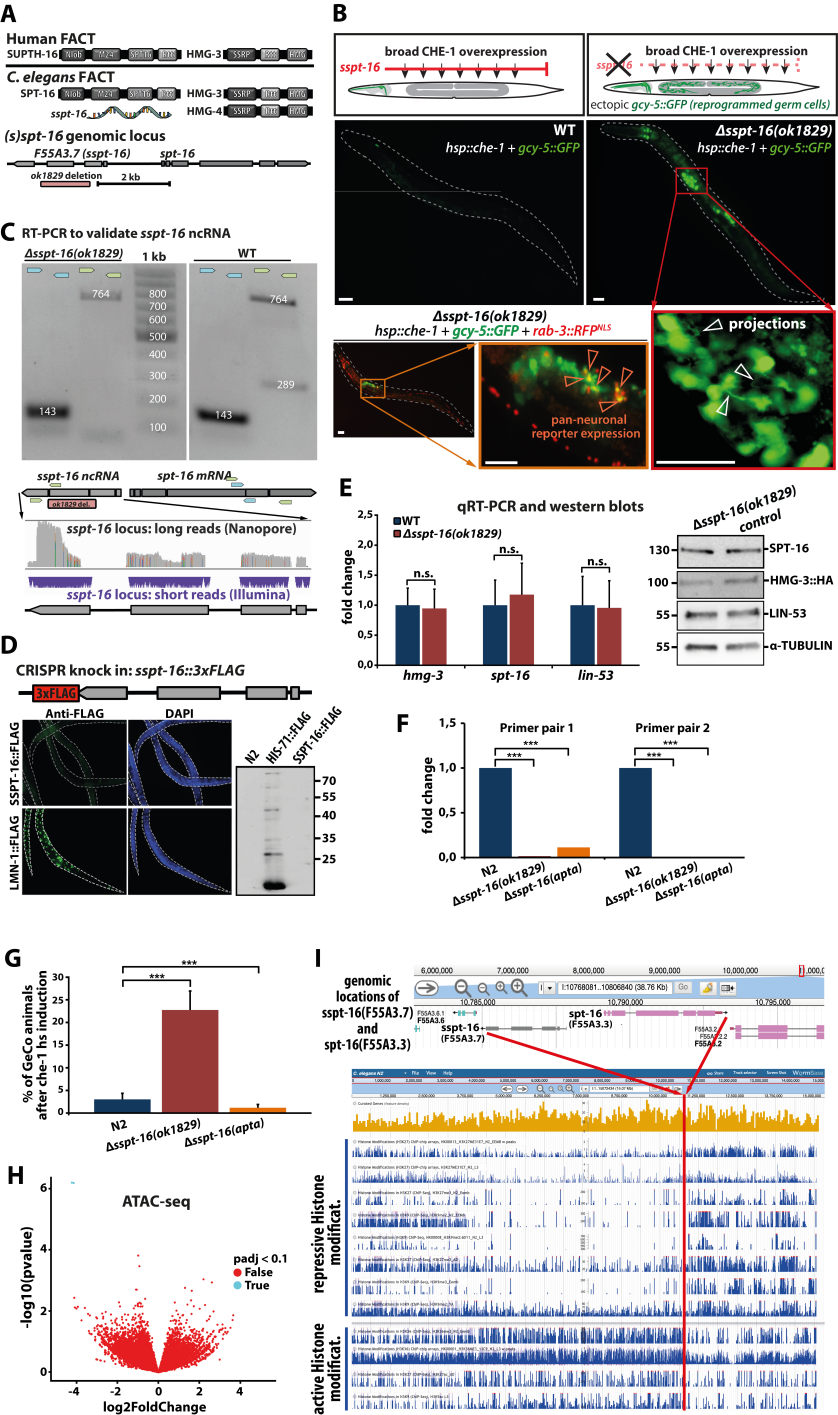
**(A)** Models of FACT subunits in *H. sapiens* (top) and *C. elegans* (middle). Conserved protein domains according to Pfam (pfam.xfam.org) and InterPro (ebi.ac.uk/interpro) are indicated. The pseudogene *sspt-16* and its mother gene *spt-16* are less than 2 kb apart from each other (bottom). The *ok1829* deletion allele of *sspt-16* is indicated in red. RNA image adapted from https://commons.wikimedia.org (CC BY-SA 3.0, free to use and change). **(B)** After broad induction of the ASE neuron specifying TF CHE-1 in L4 animals, no ectopic expression of the ASE neuron fate reporter *gcy-5p::GFP* can be detected 24 h later (top and middle left). However, in the *sspt-16(ok1829)* deletion background, ectopic *gcy-5p::GFP* expression can be detected in the germline (top and middle right). Some reprogrammed germ cells show axo-dendridic like projections (bottom right, indicated by white arrows) and some also show the pan-neuronal marker *rab-3p::NLS::tagRFP* (bottom left and middle, indicated by orange arrows). Scale bars = 20 µm. **(C)** We could verify *sspt-16* RNA transcripts through RT-PCR (top; blue primers result in 143 bp for *spt-16*, while green primers result in 764 bp for *spt-16* and 289 bp for *sspt-16*). All primers were designed at intron/exon borders, so that the expected sizes correspond to spliced mRNA or ncRNA, but not DNA. Additionally, we could detect *sspt-16* ncRNA transcripts through long-read (Nanopore) and short-read (Illumina) RNA-seq (bottom). **(D)** Using CRISPR/Cas9 gene-editing, a 3xFLAG-tag was introduced at the C-terminal end of endogenous *sspt-16* / *F55A3.7* (top). SSPT-16::3xFLAG worm lysates did not show any immunofluorescent FLAG staining, while the LMN-1::2xFLAG positive control did (bottom left). N2 (negative control), HIS-71::2xFLAG (positive control) and SSPT-16::3xFLAG worm lysates were used in a western blot. A positive band for SSPT-16::3xFLAG would have been expected at 24.3 kDa, if a tagged protein was generated from the edited *sspt-16* locus. **(E)** We checked the *sspt-16(ok1829)* deletion background for gene expression changes of the germline FACT members *spt-16* and *hmg-3,* as well as of the histone chaperone and reprogramming barrier *lin-53,* by using qPCR (left) and western blots (right), but could not detect any significant difference neither on mRNA nor on protein level for the tested genes. Error bars represent SD, n.s.: not significant according to a one-way ANOVA. **(F)** Using the CRISPR/Cas system, we generated an additional *sspt-16* deficient strain (*bar37*) by knocking in an aptamer-ribozyme sequence that depletes transcribed *sspt-16* ncRNA molecules and confirmed its functionality by qPCR using two different primer pairs which target *sspt-16* ncRNA. ***: p<0.001 according to a one-way ANOVA. **(G)** The phenotype penetrance (ectopic GFP expression in the germline after broad CHE-1 induction) was assessed for WT control, the *Δsspt-16(ok1829)* deletion strain and the *Δsspt-16(apta)* aptamer-ribozyme strain. n = 147–252; 4 biological repeats. ***: p<0.001 according to a one-way ANOVA. Error bars represent SEM. **(H)** Germline specific ATAC-seq of N2 and *Δsspt-16(ok1829)* strains revealed that there are basically no significant differences in chromatin accessibilities. Two loci that were identified as different (turquoise) were the *ok1829* deletion itself, as well as another undocumented deletion that is present in the original *ok1829* strain, so represent deletions on DNA level rather than differentially accessible chromatin. **(I)** Genomic location of s*spt-16* and *spt-16* loci showing active and repressive chromatin marks. A screenshot of JBrowse implemented in wormbase.org (WS280) is shown with publicly available tracks for repressive H3 modifications: H3K27me3 (emb/L3/adult), H3K9me2/3 (emb/L3/adult); active: H3K36me3 (emb/L3), H3K9ac (L3), H3K77ac (adult). The loci are located at the right arm of chromosome I. The red line indicates the *sspt-16* / *spt-16* location, which appears to lie within a putative chromosomal compartment border region.

## Description

FAcilitates Chromatin Transcription (FACT) is a histone chaperone complex, which is implicated in a variety of biological processes by regulating nucleosome deposition and chromatin accessibility (Orphanides *et al.* 1998; Hammond *et al.* 2017). In *C. elegans,* we previously demonstrated that FACT is a reprogramming barrier of transcription factor (TF) mediated reprogramming to neuron-like cells by safeguarding intestinal and germ cell identities (Kolundzic *et al.* 2018). Its role as a reprogramming barrier is conserved such that FACT depletion in human fibroblast culture enhanced reprogramming efficiency into both iPSCs and neurons (Kolundzic *et al.* 2018). FACT is a heterodimer consisting of two subunits: SUPT16H (suppressor of Ty 16 homolog, also known as SPT16) and SSRP1 (structure-specific recognition protein 1) in mammals (Orphanides *et al.* 1999). In *C. elegans*, SSRP1 has two homologs: HMG-3 and HMG-4, while SPT-16 is the single homolog of SPT-16 (Kolundzic *et al.* 2018)*.* The existence of the two alternative FACT subunits HMG-3 and HMG-4 is the consequence of gene duplication, resulting in two functional paralogous proteins with distinct expression patterns: HMG-3 is expressed exclusively in the germline, while HMG-4 is expressed primarily in the soma (Kolundzic *et al.* 2018). Interestingly, SPT-16 also went through a gene duplication event, resulting in the uncharacterized pseudogene *F55A3.7*, which we named *sspt-16* (short *spt-16*), because it lacks some genomic DNA pieces and an open reading frame when compared to the *spt-16* gene locus(Figure 1A).

Pseudogenes can have coding-independent regulatory functions during differentiation and tumorigenesis (Singh *et al.* 2020). A well-studied example is the pluripotency TF and tumor marker factor OCT4 with respect to its seven pseudogenes, which are differentially expressed in different tumors (Poursani *et al.* 2016). For instance, in hepatocellular carcinoma, OCT4-pg4 (OCT4 pseudogene 4) functions as a natural micro RNA sponge to regulate OCT4 expression by competing for miR-145 (Wang *et al.* 2013).

We wondered whether *sspt-16* has a function which may be related to its origin gene *spt-16* and FACT with regard to safeguarding germline identity in *C. elegans*. To address this question, we combined the deletion mutant *ok1829*, which lacks a large genomic fragment only of *sspt-16* (Figure 1A), with a strain carrying the heat-shock inducible TF CHE-1 and the ASE neuron fate reporter *gcy-5p::GFP*. We demonstrated previously that overexpressed CHE-1 induces germ cell reprogramming to ASE neuron-like cells upon HMG-3 depletion (Kolundzic *et al.* 2018) and tested whether lack of the pseudogene *sspt-16* causes a related germ cell reprogramming phenotype (Figure 1B). Indeed, after broad induction of the ASE neuron-specifying TF CHE-1 in L4 animals, ectopic expression of the ASE neuron fate reporter *gcy-5p::GFP* could be detected in the germline (assessed after 24 h) (Figure 1B). Some reprogrammed germ cells showed axo-dendritic projections (Figure 1B, bottom right), indicating that they acquired neuronal-like features. Additionally, reprogrammed germ cells also express the pan-neuronal marker *rab-3p::NLS::tagRFP*, corroborating the notion that the *sspt-16* depletion background allows conversion of germ cells to neuron-like cells upon induction of CHE-1 overexpression. Taken together, these results indicate that the pseudogene *sspt-16* exhibits a similar safeguarding function of germline identity as previously described for FACT (Kolundzic *et al.* 2018).

In order to understand how *sspt-16* contributes to safeguarding the germline we proved that it is transcribed, which we could verify by detecting *sspt-16* RNA transcripts through PCR using intron/exon border spanning primers (Figure 1C, top). Additionally, we detected *sspt-16* RNA transcripts in long-read RNA-seq by Nanopore, confirming that the transcript underwent splicing events and consists of four exons (Figure 1C, bottom). The *sspt-16* transcript should be considered as a non-coding RNA (ncRNA) because it contains two premature stop codons and a CRISPR-mediated FLAG epitope tag insertion at the presumptive C-terminus did not yield in any protein detection for immune staining and blotting (Figure 1D). Furthermore, inspection of polysome tracks provided in JBrowse (WS280) and a recent ribosome profiling study (Malone *et al.* 2017) do not indicate that a full-lenght *sspt-16 (F55A3.7)* peptide is generated. However, we cannot rule out the possibility of shorter peptides originating from the *sspt-16* locus.

Since our lab has identified other reprogramming barriers in the past (Tursun *et al.* 2011; Kolundzic *et al.* 2018; Hajduskova *et al.* 2019), we wanted to assess whether the expression levels of known factors are altered in an *sspt-16* depleted background, which could explain the resulting permissiveness for germ cell reprogramming in *ok1829* animals. We checked the *sspt-16(ok1829)* deletion background for gene expression changes of the germline FACT members *spt-16* and *hmg-3*, as well as for *lin-53,* another histone chaperone which blocks reprogramming of germ cells by promoting the formation of repressive chromatin barriers (Tursun *et al.* 2011). We performed gonad specific qPCR and whole worm western blots of WT compared to *sspt-16(ok1829)* animals, but could not detect any significant difference neither on mRNA nor on protein level for the tested factors (Figure 1E). This outcome, that lack of *sspt-16* does not affect the neighboring *spt-16* gene or other known reprogramming barrier factors, suggests that *sspt-16* is required for safeguarding germline identity through other mechanisms.

To investigate the role of *sspt-16* in safeguarding germ cells, we decided to examine whether it is sufficient to create permissiveness for germ cell reprogramming if only the *sspt-16* transcript is eliminated but not the genomic DNA sequence as in the *sspt-16(ok1829)* mutant. We therefore knocked in an aptamer-ribozyme sequence at the 3’ end of WT *sspt-16* using CRISPR/Cas. The cleavage activity of the ribozyme leads to RNA decay in absence of tetracycline (Wurmthaler *et al.* 2019). Testing by qPCR with two different primer pairs using WT animals as reference and *sspt-16(ok1829)* animals as positive control confirmed that *sspt-16* transcripts were efficiently depleted in the new aptamer-ribozyme strain *sspt-16(bar37),* from now on referred as *sspt-16(apta)* (Figure 1F). However, in contrast to the deletion allele *sspt-16(ok1829),* wherewe can consistently see a germ cell conversion phenotype in almost 25% of animals, we couldn’t detect ectopic GFP expression in the germline of *sspt-16(apta)* animals. This finding suggests that it is not only the absence of *sspt-16* ncRNA, but also the lack of genomic DNA of the *sspt-16* locus, which is required to create permissiveness for germ cell reprogramming (Figure 1G).

We speculated that the presence of the *sspt-16(ok1829)* deletion could result in altered chromatin accessibility states in germ cells thus making them more amenable for CHE-1-mediated reprogramming to neurons as previously observed upon depletion of FACT (Kolundzic *et al.* 2018). To test this hypothesis, we performed a germline-specific Assay for Transposase Accessible Chromatin with high-throughput sequencing (ATAC-seq). However, we found no significant differences in chromatin accessibility of WT vs. *sspt-16(ok1829)* germlines (Figure 1H). Only two differentially accessible loci were identified with an adjusted p value of <1, one of which represents the *ok1829* deletion itself (Figure 1H). As we could not find significant chromatin accessibility changes in *sspt-16(ok1829)* germlines, we speculate that differences at the level of chromatin modifications rather than its accessibility may be contributing to the decreased maintenance of germ cell identity in these mutants. This notion is supported by an interesting observation when revisiting publicly available data for active and repressive chromatin modifications (Figure 1I). When inspecting ChIP-seq and ChIP-chip tracks for repressive (H3K27me3 emb/L3/adult, H3K9me2/3 emb/L3/adult) and active (H3K36me3 emb/L3, H3K9ac L3, H3K77ac adult) H3 modifications, it appears that the *sspt-16* / *spt-16* locus lies within a region of the right arm of chromosome I, which delineates a boundary for repressive and active chromatin. Thus, we speculate that the lack of genomic DNA within the *sspt-16* locus may lead to a boundary weakening, which could cause spreading of active chromatin and thereby allow activation of ectopic gene expression. Yet, the exact mechanism of how lack of the *sspt-16* locus causes permissiveness for germ cell reprogramming remains to be determined and future investigation should focus on changes of chromatin modifications in the *sspt-16* deletion mutant.

## Methods

*C. elegans* strains and maintenanceAll strains used were grown in nematode growth medium (NGM) at 15°C as previously described (Brenner 1974). Heat-shocking of strains was performed at 37° C for 30 min following incubation at 25° C O/N.

RNA-seqLibrary preparation for RNA sequencing was carried out using NEXTFLEX Rapid Directional RNA-Seq Kit 2.0 (Bioo Scientific) and SQK-DCS109/EXP-NBD104 (Nanopore) according to the manufacturer’s instructions. Libraries were sequenced using paired‐end sequencing length of a 100 nucleotides on a HiSeq 4000 machine (Illumina) and Minion (Nanopore).

Gonad dissectionGonads were dissected as previously described (Jones *et al.* 1996).

Gonad specific ATAC-seqEach replicate of dissected gonads (50 gonad arms in 20 µl ddH_2_O in a 1.5 ml Eppendorf tube) was filled up with 700 µl of freshly prepared and precooled buffer A (15 mM Tris–HCl pH 7.5/2 mM MgCl2/0.34 M sucrose/0.15 mM spermine/0.5 mM spermidine/1 mM DTT/0.5 mM PMSF/0.25% NP40/0.1% Triton X-100) (Ooi *et al.* 2010) and subjected to 30 strokes of a tight fitting plastic pestle to open up the gonads and to isolate the nuclei. Subsequently, the pestle was rinsed with 300 µl of buffer A, each sample was spun for 5 min (5000 g/4° C) and the supernatant was discarded. The in this way purified nuclei were tagmented using the Nextera DNA Flex Library Prep Kit (Illumina) according to the manufacturer’s instructions, adding additional PBS and Tween-20 to the tagmentation buffer as described by (Corces *et al.* 2017) and sequenced using paired-end-sequencing length of 75 nucleotides on a HiSeq4000 machine (Illumina).

## Reagents

List of strains

**Table:** 

**Name**	**Genotype**	**Availability**
N2	*Caenorhabditis Elegans*	CGC
RB1524	*F55A3.7(ok1829) I.*	CGC
BAT28	*otIs305 [hsp-16.2p::che-1::3xHA, rol-6(su1006)], ntIs1 [gcy-5p::GFP, lin-15(+)] V.*	upon request
BAT372	*F55A3.7(ok1829) I; otIs305 [hsp-16.2p::che-1::3xHA, rol-6(su1006)], ntIs1 [gcy-5p::GFP, lin-15(+)] V.*	upon request
BAT748	*barEx308 [hsp-16.2::H3_S10E::2xFLAG, hsp-16.2::His-71_S10E::2xFLAG, myo-2p::mCherry], otIs305 [hsp-16.2p::che-1::3xHA, rol-6(su1006)], ntIs1 [gcy-5p::GFP, lin-15(+)] V.*	upon request
BAT1468	*barIs165 [myo-3p::lmn-1T40I::2xFLAG::SL2::NLS::tagRFP]*	upon request
BAT1594	*F55A3.7(bar23[F55A3.7::3xFLAG]) I.*	upon request
BAT1560	*hmg-3(bar24[hmg-3::3xHA]) I.*	upon request
BAT1749	*hmg-3(bar24[hmg-3::3xHA]) I; F55A3.7(ok1829) I.*	upon request
BAT2099	*F55A3.7(bar37[F55A3.7::aptamer]) I; otIs305 [hsp-16.2p::che-1::3xHA, rol-6(su1006)], ntIs1 [gcy-5p::GFP, lin-15(+)] V.*	upon request
BAT2192	*F55A3.7(ok1829) I; otIs355 [rab-3::NLS::TagRFP]; otIs305 [hsp-16.2p::che-1::3xHA, rol-6(su1006)], ntIs1 [gcy-5p::GFP, lin-15(+)] V.*	upon request

List of (q)RT-PCR primers

**Table:** 

**Forward primer**	**Sequence**	**Reverse primer**	**Sequence**	**Target Gene**
oBT504	GTGTGGGACCTATCTAAGA	oBT223	TTACTGTTGTCTCTCTACCAC	*lin-53*
oBT823	GGATCGTTGGAGGCTCATACT	oBT824	TCATTATCAGATTCAGCATTCAGG	*spt-16* and *sspt-16*
oBT827	CATTTTCGAGTTTGGGAAGG	oBT828	CCATTGAAATAGTCGAGTTGT	*hmg-3*
oBT839	GGAGGCCCACACTAATGGATTTAGATACACATC	oBT840	CTGGATTCTTGAGATGGAAGTGC	*spt-16*
oBT863	CTGCTGGACAGGAAGATTACG	oBT864	CTCGGACATTCTCGAATGAAG	*cdc-42*
oBT4011	GCTGAACAACGGGAGAAGGA	oBT4012	ACGCGAAAATTGGCTGTACAAA	*sspt-16*

List of antibodies

**Table:** 

**Primary AB**	**Host & Clonality**	**Dilution**	**Company**	**Secondary AB**
Anti-α-tubulin	mouse, mono	1:10000	Sigma	Anti-mouse-HRP, 1:10000, Santa Cruz Biotechnology Inc.
Anti-FLAG	mouse, mono	1:1000	Sigma	Anti-mouse-HRP, 1:10000, Santa Cruz Biotechnology Inc. or Anti-mouse AlexaFluor488, 1:1500, Molecular Probes
Anti-HA-HRP	chicken, poly	1:1000	Abcam	–
Anti-LIN-53	rabbit, mono	1:2000	Pineda	Anti-rabbit-HRP, 1:10000, Santa Cruz Biotechnology Inc.
Anti-SPT-16	guinea pig, mono	1:2000	Pineda	Anti-guinea pig-HRP, 1:10000, Santa Cruz Biotechnology Inc.
